# Agreement between cause of death assignment by computer-coded verbal autopsy methods and physician coding of verbal autopsy interviews in South Africa

**DOI:** 10.1080/16549716.2023.2285105

**Published:** 2023-12-01

**Authors:** Pam Groenewald, Jason Thomas, Samuel J Clark, Diane Morof, Jané D. Joubert, Chodziwadziwa Kabudula, Zehang Li, Debbie Bradshaw

**Affiliations:** a Burden of Disease Research Unit, South African Medical Research Council, Cape Town, South Africa; b Department of Sociology, The Ohio State University, Columbus, Ohio, USA; c Division of Global HIV & TB, Centers for Disease Control and Prevention, Durban, South Africa; d United States Public Health Service Commissioned Corps, Rockville, Maryland, USA; e MRC/Wits Rural Public Health and Health Transitions Research Unit (Agincourt), University of Witwatersrand, Johannesburg, South Africa; f Department of Statistics, University of California Santa Cruz, Santa Cruz, California, USA; g Division of Public Health Medicine, School of Public Health, University of Cape Town, Cape Town, South Africa

**Keywords:** Physician-coded, computer-coded, verbal autopsy, agreement

## Abstract

**Background:**

The South African national cause of death validation (NCODV 2017/18) project collected a national sample of verbal autopsies (VA) with cause of death (COD) assignment by physician-coded VA (PCVA) and computer-coded VA (CCVA).

**Objective:**

The performance of three CCVA algorithms (InterVA-5, InSilicoVA and Tariff 2.0) in assigning a COD was compared with PCVA (reference standard).

**Methods:**

Seven performance metrics assessed individual and population level agreement of COD assignment by age, sex and place of death subgroups. Positive predictive value (PPV), sensitivity, overall agreement, kappa, and chance corrected concordance (CCC) assessed individual level agreement. Cause-specific mortality fraction (CSMF) accuracy and Spearman’s rank correlation assessed population level agreement.

**Results:**

A total of 5386 VA records were analysed. PCVA and CCVAs all identified HIV/AIDS as the leading COD. CCVA PPV and sensitivity, based on confidence intervals, were comparable except for HIV/AIDS, TB, maternal, diabetes mellitus, other cancers, and some injuries. CCVAs performed well for identifying perinatal deaths, road traffic accidents, suicide and homicide but poorly for pneumonia, other infectious diseases and renal failure. Overall agreement between CCVAs and PCVA for the top single cause (48.2–51.6) indicated comparable weak agreement between methods. Overall agreement, for the top three causes showed moderate agreement for InterVA (70.9) and InSilicoVA (73.8). Agreement based on kappa (−0.05–0.49)and CCC (0.06–0.43) was weak to none for all algorithms and groups. CCVAs had moderate to strong agreement for CSMF accuracy, with InterVA-5 highest for neonates (0.90), Tariff 2.0 highest for adults (0.89) and males (0.84), and InSilicoVA highest for females (0.88), elders (0.83) and out-of-facility deaths (0.85). Rank correlation indicated moderate agreement for adults (0.75–0.79).

**Conclusions:**

Whilst CCVAs identified HIV/AIDS as the leading COD, consistent with PCVA, there is scope for improving the algorithms for use in South Africa.

## Introduction

South Africa boasts a robust civil registration and vital statistics system, registering over 90% of adult deaths. Nevertheless, the accuracy of cause of death (COD) data is questionable, plagued by significant underreporting of HIV/AIDS-related deaths [1], inaccuracies in injury cause profiles [2], and a high proportion of deaths coded to unusable International Classification of Diseases (ICD) codes [3]. The National Cause of Death Validation (NCODV 2017/18) project aimed to validate official reported COD against physician-coded verbal autopsy (PCVA) data and medical/forensic records to estimate accurate cause-specific mortality fractions for South Africa [4]. This provided the first national sample of VAs as use in South Africa has been limited to health and demographic surveillance sites (HDSS) [5].

VA is traditionally used in areas with limited medical certification of COD. It involves a structured interview with close relatives or caregivers about the symptoms and signs leading to death with a physician assigning a COD (PCVA) based on information provided [6]. However, the resource-intensive nature of setting up access to narratives and VA responses and physician coding makes it less feasible in low- and middle-income countries. The development of automated computer-coded VA (CCVA) methods has increased the possibility for more routine use of VA [7–10]. In South Africa, the InterVA algorithm has been validated against PCVA for VA data in two HDSS sites. The Agincourt study demonstrated InterVA-3’s utility at the population level but not the individual level [11], while the Africa Centre study revealed substantial overall agreement of 79% between PCVA and InterVA coding at the burden of disease main cause group level [12]. Whilst there is ongoing debate about the validity of PCVA as a reference standard for assessing cause of death [6,13], the reliability of the proposed alternatives, medical certification of cause of death (MCCD) or autopsy have also been challenged, and may not be available in some settings [6,13]. PCVA has been used for many years in South Africa, has successfully tracked trends in cause of death during the HIV epidemic and the health transition and there remains confidence in the COD assigned by PCVA in the country.

Conceptually, COD assignment for CCVA consists of three key components: the VA data, the symptom-cause information (SCI), and the deterministic or probabilistic algorithm that combines the first two components to rank causes for each death. The SCI depicts how VA symptoms and causes are related. However, comparing the performance of the CCVA algorithms reported in the literature is challenging due to disparate datasets (the range and frequencies of conditions used for the assessment affects the measures of performance) and where datasets are comparable, the symptom cause information (SCI) for certain CCVA algorithms is closely linked to one specific limited reference dataset [8]. The choice of SCI can impact CCVA method performance as much as or even more than algorithm logics [14].

In the NCODV 2017/18 project an objective was to assess the agreement between CODs assigned by CCVA algorithms and PCVA, without additional medical record information. Three CCVA methods – InterVA-5 [15], InSilicoVA [8] and Tariff 2.0 [10] – were employed for COD assignment, enabling a comparison of their performance with PCVA in assigning a COD at individual level and characterizing cause-specific mortality fractions (CSMFs) at population level.

## Methods

### Setting

In 2017 the population of South Africa was 56 million people, of which 51% were women. The country faces a quadruple burden of disease [16] with poverty-related diseases, a massive HIV/AIDS and TB burden, and high rates of non-communicable diseases, violence and injuries. A nationally representative sample of registered deaths based on prospective recruitment of next-of-kin was collected from 27 sub-districts across the country [4]. Recruitment was done through funeral undertakers and Department of Home Affairs offices and was very difficult outside the pilot district. The recruitment rate was poor with the possibility of bias in the overall COD profile. Between 1 July 2017 and 30 April 2018 trained fieldworkers collected VA data from next-of-kin using the 2016 WHO VA instrument [17].

### Physician assignment of cause of death

For the NCODV 2017/18 project we recruited physician coders who had clinical experience in the public health care system and were familiar with the common diseases in South Africa. Their training included the public health importance of causes of death, ICD-10 guidelines for medical certification of COD (MCCD) and interpretation of verbal autopsy narratives and interviews from the WHO2016 VA instrument. In addition, they received training on the content and technical aspects of the electronic review questionnaire. They were required to demonstrate their competence in MCCD and successfully review 10 practice VA case scenarios and a competency test. Out of the ~ 100 physicians trained only those who demonstrated their competence were asked to review the study records. Seventy-five clinicians were appointed to review records. Five of these clinicians, who demonstrated superior competence, were designated as quality assessment reviewers. Each VA record was independently reviewed by two clinicians and a COD sequence and contributing COD certified according to WHO ICD-10 guidelines [18]. The quality assessment team assessed the reviewed cases. Where there was disagreement in the assigned COD, the two clinicians were notified and asked to reach consensus. Where they were unable to reach consensus the quality assessment team reviewed the case and assigned a cause of death. In 3115 (57.5%) cases there was agreement on the COD between independent reviewers whilst in 1744 (42.8%) cases the independent reviewers needed to reach consensus. Only 10.3% of cases needed referral to the QA team. The certified COD were coded to ICD-10 using the automated coding software IRIS [19].

### CCVA methods

In this sub-analysis of the NCODV 2017/18 project, three CCVA methods assigned a COD to each VA record. InterVA-5 [15] is a deterministic algorithm based on the 2016 WHO VA instrument. This uses a physician-provided, pre-defined SCI that describes the conditional probability of each symptom given each cause. COD assignments are obtained based on a modified Naïve Bayes classifier. InSilicoVA [9] is a Bayesian hierarchical cause-of-death assignment method that uses the same SCI as InterVA-5, but jointly estimates both individual- and population-level cause-of-death distributions under a probabilistic framework. The Tariff 2.0 algorithm [10] is another deterministic algorithm based on the SCI derived from the Population Health Metrics Research Consortium (PHMRC) Gold Standard VA Validation Study [20]. Tariff 2.0 is a score-based method that compares different causes of death based on the observed symptoms. Tariff 2.0 differs from the first two methods in that it does not explicitly compute a continuous indicator of the strength of the relationship between each cause and a given VA death (a propensity score is given for InterVA-5 and a probability distribution for InSilicoVA). Instead, it produces a ranking of causes for each death. InterVA-5 and Tariff 2.0 both assign ‘indeterminate’ (or undetermined) to COD from VAs with comparatively less information. For Tariff2.0 a ‘likelihood’ Tariff 2.0 score is calculated and if the value exceeds a threshold, then the nominated cause is assigned. Where the score does not exceed the threshold, the cause is indeterminate. For InterVA-5 the cut-off is a likelihood of 0.4 [21]. For Tariff 2.0, if the score was below either the cause-specific or the absolute thresholds, the cause was classified as indeterminate as no cut off is specified [10]. InSilicoVA assigns a cause with uncertainty to all VA deaths; deaths with less information have more uncertainty.

The assignment of COD was implemented with the R packages for InterVA-5 [22] and InSilicoVA [23] through the standardized openVA toolkit [24]. COD assignment via Tariff 2.0 2.0 was conducted using the SmartVA-Analyze tool [25].

### NCODV 2017/18 verbal autopsy dataset

The planned sample size of verbal autopsies was 13 000 VA records for deaths of all ages, both sexes and occurring in and out of health facilities across South Africa.

### Analysis cause list

The PCVA ICD-10 codes were mapped to the WHO VA COD list [26] to enable comparison between PCVA and CCVA. The WHO VA cause list was mapped to a shorter list of 25 causes (25-cause list) as some causes had very few cases and comparison was unlikely to be meaningful (see Table S1).

### Performance metrics for cause of death assignment

Prior to investigation of the agreement on cause-assignment between PCVA and CCVAs the cause-specific mortality fractions (CSMF) produced by CCVA and PCVA were compared by broad cause group and by the 25-cause list, including undetermined causes. The undetermined causes identified through the PCVA method were then purposefully removed before investigating the performance of the CCVA methods for COD assignment against the COD assigned by PCVA.

Thereafter performance metrics were used to assess agreement at individual and at population level. Positive predictive value (PPV), sensitivity, overall agreement, kappa statistic, and chance corrected concordance (CCC) were used to assess agreement at individual level, using the most likely COD assigned by the algorithms. Agreement at population level was assessed using CSMF accuracy, and Spearman’s rank correlation. The performance of algorithms as measured using overall agreement, kappa, CCC, CSMF accuracy, and rank correlations was compared between age and sex subgroups as well as for in and out of facility.

In addition, the overall agreement using the top three causes for InterVA-5 (ignoring the cut-off of 0.4) and InSilicoVA was compared with the overall agreement based on the most likely cause. This was the percentage of deaths for which the PCVA COD is included in one of the three most likely causes identified by the CCVAs. Since Tariff 2.0 only produces one most likely cause per record, it was not included in this analysis.

### Individual level metrics

**Positive Predictive Value** measures the percentage of deaths CCVA assigned to a particular COD that were also assigned by PCVA to the same cause. When CCVA makes this prediction, PPV indicates how often this prediction is correct (i.e. is the particular cause assigned by PCVA). While useful, this metric tends to indicate better performance when the prevalence of a COD increases (as the chance for false positives decreases).

PPV for cause *j* is calculated as follows:

$$PPV_{j}=\;\frac{TP_{j}}{TP_{j}\;+FP_{j}}$$

where *TP*_*j*_ is true positives (where CCVA COD is the same as the specific COD assigned by PCVA) and *FP*_*j*_ is false positives (where CCVA assigned the specific COD, but PCVA assigned a different COD).

**Sensitivity** measures the percentage of deaths identified by CCVA due to a particular COD out of the total deaths for that cause as assigned by PCVA. This gives an indication of how sensitive CCVA is at identifying the correct COD (COD assigned by PCVA) and if the CCVA is measuring what it is supposed to measure, a concept typically referred to as validity. Simply put, when PCVA assigns cause X, how often does CCVA provide the same cause assignment.

Sensitivity for cause *j* is calculated as follows:

$$Sensitivity_{j}=\;\frac{TP_{j}}{TP_{j}\;+FN_{j}}$$

where *TP*_*j*_ is true positives (where CCVA assigns the same specific COD as assigned by PCVA) and *FN*_*j*_ is false negatives (where CCVA assigns a different COD to the specific COD assigned by PCVA).

**Chance-corrected concordance** [27] is similar to PPV but accounts for correct COD assignment made purely by chance. CCC for cause *j* is calculated as follows:

$$CCC_{j}=\frac{\left(\frac{TP_{j}}{TP_{j}+FN_{j}}\right)-\left(\frac{1}{C}\right)}{1-\left(\frac{1}{C}\right)}$$

where *TP*_*j*_ is true positives, *FN*_*j*_ is false negatives and *C* is the number of causes.

**Overall Agreement** is the percentage of deaths for which the PCVA and CCVA assign the same cause.

**Cohen’s kappa** [28] accounts for the fact that the PCVA and CCVA agree on some assigned COD by chance. It can range from −1 to + 1. Cohen’s kappa is:

$$k=\;\left(p_{o}-p_{e}\right)\;/\;\left(1\;-p_{e}\right)$$

where:

- *p*_*o*_: relative observed agreement among COD assigned by PCVA and CCVA
- *p*_*e*_: hypothetical probability of chance agreement.

The kappa statistic values with the corresponding percentage of data that agree were used to define thresholds for level of agreement based on recommendations by McHugh (2012) [29] and shown in Table 1.

**Table 1.** Level of agreement thresholds for kappa statistic.

Value of Kappa	Level of Agreement	% Data that agree
-.01 – .20	None	0–4%
.21 – .39	Minimal	4–15%
.40 – .59	Weak	15–35%
.60 – .79	Moderate	35–63%
.80 – .90	Strong	64–81%
Above .90	Almost Perfect	82–100%

Source: McHugh [29].

### Population level metrics

**CSMF accuracy** [30] quantifies how closely the CCVA CSMF values approximate the PCVA CSMF values.

$$CSMF\;accuracy=1-\frac{\sum_{j=1}^{k}|{CSMF}_{j}^{true}-{CSMF}_{j}^{pred}|}{2\left(1-Minimum\left({CSMF}_{j}^{true}\right)\right)}$$

CSMF accuracy ranges from 0 to 1, with 0 being the worst case and 1 being perfect alignment between the CCVA CSMF and PCVA CSMF.

Flaxman et al. [30] proposed a **chance corrected CSMF accuracy metric**, which is defined as a deterministic linear transformation of the CSMF accuracy:

$$\begin{array}{rl} & Chance-corrected\;CSMF\;accuracy\\ & \;\;\;\;\;\;\;\;\;\;\;=\left(CSMF\;accuracy-0\cdot 632\right)/\left(1-0\cdot 632\right) \end{array}$$

We omit the metric here as it provides the same information as the CSMF accuracy.

**Spearman’s rank correlation coefficient** [31] is another population-level agreement metric which measures the similarities between the rankings of the causes produced by CCVA and the actual ranking (as produced by PCVA). It is computed as follows:

$$Rho=corr\left(Rank\left(CSMF{\;}^{\text{\textbackslash{}\textasciicircum{}}}\;pred\right),Rank\left(CSMF{\;}^{\text{\textbackslash{}\textasciicircum{}}}\;true\right)\right)$$

where Rank (CSMF) is the rank of CSMFs, i.e. the cause with the largest CSMF receives a rank of 1 and the cause with smallest CSMF receives a rank of 25. Unlike the CSMF accuracy, which evaluates the absolute differences between the estimated and true CSMFs, the rank correlation coefficient is less sensitive to a large CSMF due to a single cause.

Due to the complexity of the algorithms, 95% confidence intervals were not calculated for Chance corrected concordance and CSMF accuracy. Confidence intervals were calculated for the sensitivity and PPV, assuming simple proportions to get a sense of the variability.

## Ethics

The project protocol was reviewed by the SAMRC Ethics committee and approved on 27 June 2017 (EC004–2/2017). This project was reviewed in accordance with CDC human research protection procedures and was determined to be research, but CDC investigators did not interact with human subjects or have access to identifiable data or specimens for research purposes (8 April 2017).

## Results

### Characteristics of the sample

A total of 5386 verbal autopsy records from 2579 (47.9%) female and 2807 (52.1%) male deaths, coded by both PCVA and CCVA, were available for analysis. There were 102 neonates (0-27 days), 204 children (28 days − 11 years), 2018 adults (12–49 years) and 3062 elders (50 + years). The median age was 54 years (IQR: 36–69). A total of 2926 had died in a health facility with the remaining 2460 dying outside a health facility.

The PCVA CSMF for broad COD groups (including undetermined causes) showed the typical quadruple burden seen in South Africa, with HIV/AIDS & TB deaths accounting for 28.1%, other infectious, maternal, perinatal and nutritional diseases 8.8%, non-communicable diseases 34.6%, and injuries 12.6%. There were no stillbirths in this sample of VAs. The proportion of deaths with ill-defined causes (*N* = 852) assigned by PCVA was high at 15.8%. These records were excluded for the agreement analyses. Tariff 2.0 assigned 48.0% of the PCVA undetermined COD cases to undetermined, followed by 12.3% to diabetes mellitus (Figure S1). InterVA-5 and InSilicoVA assigned a large proportion of these to cardiac causes (38.0% and 26.3% respectively).

### Comparison of cause specific mortality profiles across PCVA and CCVAs

**Figure 1.**
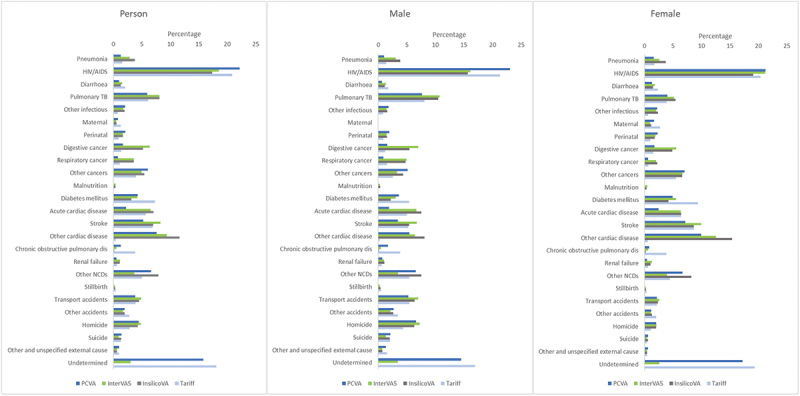
Cause-specific mortality fractions for PCVA, InterVA-5, InSilicoVA and Tariff 2.0 for 25-cause list including undetermined causes for persons, males and females, NCODV SA 2017/18.

A comparison of the CSMF (25-cause list) between PCVA and CCVAs for persons, males and females showed differences (Figure 1). Whilst HIV CSMFs for PCVA (0.22) and Tariff 2.0 (0.21) for persons were close, those for InterVA-5 (0.19) and InSilicoVA (0.17) were lower, Figure 1. Pulmonary TB CSMFs for females for PCVA (0.04) and Tariff 2.0 (0.04) were similar, with InterVA-5 (0.05) and InSilicoVA (0.05) being higher. For pulmonary TB in males InterVA-5 (0.11) and InSilicoVA (0.11) were similar followed by Tariff 2.0 (0.08) and PCVA (0.08). Tariff 2.0 had the highest diabetes mellitus CSMF (0.07) in persons (data not shown), followed by PCVA and InterVA-5 (0.04) followed by InSilicoVA (0.03). Acute cardiac disease CSMFs in persons (data not shown) were lowest for PCVA (0.02) and highest for InterVA-5 and InSilicoVA (0.07). Digestive cancer in persons (data not shown) was much higher for InterVA-5 (0.06) and InSilicoVA (0.05) compared with PCVA (0.01) and Tariff 2.0 (0.01). Lung cancer CSMF in persons was highest for PCVA (0.06) than InterVA-5 (0.04) and InSilicoVA (0.04) with much lower values produced by Tariff 2.0 (0.01). Maternal and COPD were much higher for Tariff 2.0 (0.01 and 0.04 respectively) compared with PCVA (0.01; 0.01), InterVA-5 (0.01; 0.01) and InSilicoVA (0.01; 0.001). Undetermined causes were slightly higher in Tariff 2.0 (0.18) than PCVA (0.16).

PCVA – physician coded verbal autopsy, NCD – non-communicable diseases, TB – tuberculosis, COPD – Chronic obstructive pulmonary disease.

Although no stillbirths were in the sample, all algorithms allocated some deaths to stillbirths, see Figure S2(a) (CSMF for neonates by the 25-cause list). The CSMF for neonates using the WHO VA cause list is shown in Figure S2(b).

### Individual level agreement

**Figure 2.**
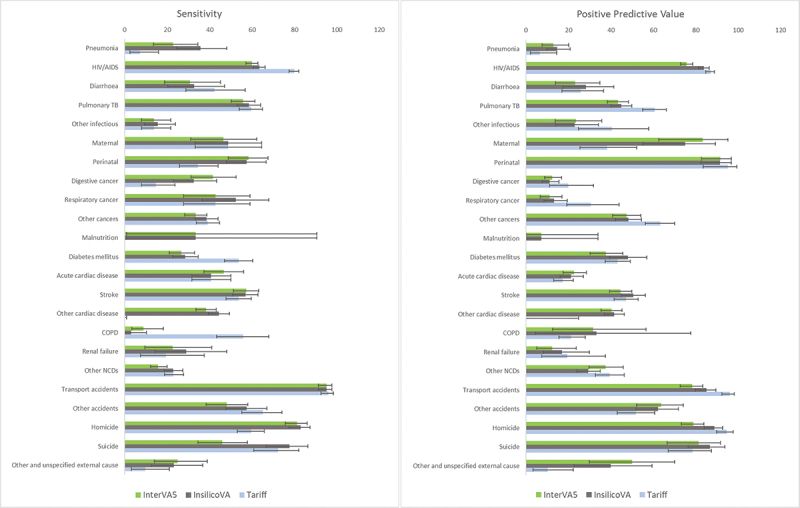
Sensitivity and PPV of computer-coded verbal autopsy methods versus physician-coded verbal autopsy reference standards for cause of death, 25-cause list, NCODV SA 2017/18.

After exclusion of the PCVA undetermined causes (*N* = 852) 4534 records were available for agreement analyses. The sensitivity and PPV by cause for each algorithm are shown in Figure 2. Overlapping confidence intervals for PPV and sensitivity suggest that the performance of all CCVAs is comparable for most causes of death, except HIV/AIDS, TB, maternal, diabetes mellitus, other cancers, and some injuries where there were differences. Tariff 2.0 performed best at identifying HIV/AIDS deaths (sensitivity 79.9%; PPV 87.0%), had significantly higher sensitivities for diabetes mellitus (sensitivity 53.7%) and COPD (sensitivity 55.9%) and significantly higher PPV for pulmonary TB (60.7%), other cancers (63.4%) and transport injuries (96.1%). InterVA-5 and InsilicoVA had significantly higher PPV for maternal deaths than Tariff 2.0.

Tariff 2.0 does not assign causes to malnutrition or other cardiac disease.

NCD – non-communicable diseases, TB – tuberculosis, COPD – Chronic obstructive pulmonary disease.

All algorithms performed very well at identifying transport accidents with sensitivities of over 95%. Both InterVA-5 and InSilicoVA performed well at identifying homicide (sensitivity 81.3%; 82.9%) and InSilicoVA reasonably well at identifying suicide (sensitivity 77.6%). All algorithms were particularly poor at identifying deaths due to pneumonia, other infectious diseases, and renal failure.

Overall agreement, kappa statistic, CCC by age, sex and in- or out-of-facility deaths are displayed in Table 2. InSilicoVA had the highest overall agreement with PCVA for all groups except adults, males and in-facility deaths where Tariff 2.0 performed better. InSilicoVA overall agreement for the total sample was 51.6%, 62.3% for adults and 79.3% for neonates. Tariff 2.0 had the highest overall agreement for adults (65.4%) and males (55.3%) and lowest for neonates (55.3%) and children (55.3%). InterVA-5 overall agreement was slightly lower than InSilicoVA for all groups. Overall agreement for children was below 40.6% for all algorithms. The kappa statistic demonstrated weak to minimal agreement for all algorithms and all groups with Tariff 2.0 having the highest Kappa for adults (0.56) and males (0.50). For CCC, agreement was below 0.4 for all algorithms and groups.

**Table 2.** Overall agreement, kappa, chance corrected concordance and CSMF accuracy between cause of death assignment by PCVA and 3 CCVA methods, 25 cause list, NCODV SA 2017.

Verbal autopsy coding method	Individual level agreement				Population level agreement	
	Overall Agreement Top cause (95% CI)	Overall Agreement Top 3 causes (95% CI)	Kappa Top cause (95% CI)	Chance corrected concordance (CCC) Top cause	Cause specific mortality fraction (CSMF) Accuracy	Spearman Rank correlation (95% CI)
**Total sample *N* = 4534**						
InterVA-5	48.2	70.9	0.43	0.39	0.81	0.64 (0.62–0.65)
	(46.7–49.7)	(69.6–72.2)	(0.42–0.44)			
InSilicoVA	51.6	73.8	0.47	0.42	0.84	0.68 (0.67–0.70)
	(50.2–53.1)	(72.5–75.1)	(0.46–0.48)			
Tariff 2.0	51.2	*	0.46	0.38	0.82	0.66 (0.65–0.68)
	(49.8–52.7)		(0.45–0.47)			
**Neonate (0–27 days) *n* = 82**						
InterVA-5	78.5	78.0	−0.05	0.13	0.90	0.02 (−0.02–0.24)
	(67.5–86.4)	(67.5–86.4)	(−0.14–0.04)			
InSilicoVA	79.3	79.3	−0.04	0.13	0.84	0.02 (−0.02–0.24)
	(68.9–87.4)	(68.9–87.4)	(−0.14–0.05)			
Tariff 2.0	47.6	*	0.01	0.06	0.83	−0.06 (−0.27–0.16)
	(36.4–58.9)		(−0.04–0.06)			
**Child (28 days − 11 years) *n* = 165**						
InterVA-5	36.4	50.3	0.32	0.32	0.66	0.43 (0.30–0.55)
	(29.0–44.2)	(42.4–58.2)	(0.28–0.36)			
InSilicoVA	40.6	56.4	0.36	0.40	0.64	0.65 (0.55–0.73)
	(33.0–48.5)	(48.4–64.1)	(0.32–0.40)			
Tariff 2.0	28.5	*	0.24	0.20	0.66	0.50 (0.37–0.60)
	(21.7–36.0)		(0.20–0.28)			
**Adult (12–49 years) *n* = 1812**						
InterVA-5	58.3	77.2	0.49	0.34	0.80	0.75 (0.73–0.77)
	(56.0–60.6)	(75.1–79.1)	(0.47–0.51)			
InSilicoVA	62.3	79.7	0.54	0.37	0.84	0.79 (0.77–0.81)
	(60.0–64.5)	(77.8–81.5)	(0.52–0.56)			
Tariff 2.0	65.4	*	0.56	0.38	0.89	0.75 (0.73–0.75)
	(63.2–67.6)		(0.55–0.58)			
**Elder (50+ years) *n* = 2475**						
InterVA-5	40.6	67.5	0.35	0.34	0.80	0.47 (0.44–0.50)
	(38.7–42.6)	(65.6–69.4)	(0.34–0.36)			
InSilicoVA	43.7	70.4	0.38	0.37	0.83	0.51 (0.48–0.54)
	(41.7–45.7)	(68.6–72.2)	(0.37–0.40)			
Tariff 2.0	42.5	*	0.38	0.39	0.76	0.55 (0.52–0.58)
	(40.5–44.5)		(0.37–0.39)			
**Male *n* = 2400**						
InterVA-5	47.7	71.1	0.43	0.39	0.76	0.68 (0.66–0.70)
	(45.7–49.7)	(69.3–72.9)	(0.41–0.44)			
InSilicoVA	52.0	74.1	0.47	0.43	0.80	0.73 (0.71–0.73)
	(50.0–54.0)	(72.3–75.9)	(0.46–0.48)			
Tariff 2.0	55.3	*	0.50	0.39	0.84	0.71 (0.69–0.73)
	(53.2–57.3)		(0.49–0.52)			
**Female *n* = 2134**						
InterVA-5	48.8	70.7	0.43	0.38	0.87	0.57 (0.54–0.60)
	(46.6–50.9)	(68.7–72.6)	(0.41–0.44)			
InSilicoVA	51.2	73.4	0.46	0.40	0.88	0.61 (0.59–0.64)
	(49.1–53.4)	(71.5–75.2)	(0.44–0.47)			
Tariff 2.0	46.7	*	0.41	0.34	0.79	0.60 (0.57–0.63)
	(44.6–48.9)		(0.40–0.43)			
**Died in health facility *n* = 2591**						
InterVA-5	47.4	71.2	0.41	0.38	0.80	0.57 (0.54–0.59)
	(45.4–49.3)	(69.4–72.9)	(0.40–0.42)			
InSilicoVA	50.6	73.5	0.45	0.43	0.82	0.63 (0.60–0.65)
	(48.7–52.6)	(71.8–75.2)	(0.43–0.46)			
Tariff 2.0	51.6	*	0.45	0.36	0.82	0.65 (0.63–0.67)
	(49.7–53.5)		(0.44–0.46)			
**Died out of health facility *n* = 1943**						
InterVA-5	49.3	70.6	0.45	0.39	0.82	0.71 (0.69–0.73)
	(47.1–51.6)	(68.5–72.6)	(0.44–0.46)			
InSilicoVA	53.0	74.1	0.49	0.41	0.85	0.74 (0.72–0.76)
	(50.7–55.2)	(72.1–76.0)	(0.48–0.50)			
Tariff 2.0	50.7	*	0.47	0.40	0.81	0.66 (0.64–0.69)
	(48.5–53.0)		(0.46–0.48)			

CI – confidence interval.

*Tariff 2.0 only produces the most likely cause for each record thus could not be included in the Top 3 causes overall agreement analysis.

### Population level agreement

CSMF accuracy was above 0.80 for all algorithms for the total sample, neonate and adult groups, in males for InSilicoVA and Tariff 2.0 and in females for InSilicoVA and InterVA-5 suggesting moderate to strong agreement (Table 2). For CSMF accuracy, InterVA-5 had the highest agreement (0.90) for neonates, Tariff 2.0 the highest agreement for adults (0.89) and males (0.84), and InSilicoVA the highest agreement for females (0.88), elders (0.83) and out of health facilities (0.85). The CSMF accuracy was lowest for the child group for all algorithms (0.64–0.66). The Spearman’s rank correlation was highest for adults at 0.8 for InSilicoVA and Tariff 2.0 and was lowest for neonates for all algorithms (Table 2).

## Discussion

This study highlights the comparable performance of CCVA methods in identifying leading COD at population level. However, improvement in the SCI is required before CCVA can replace PCVA for individual COD assignment. According to the clinician reviewers in this study the VA narrative provided important information for PCVA, however this is not routinely included in any of the CCVA methods.

A reasonable level of agreement (>75%) in CSMF accuracy between PCVA and CCVAs was evident at population level, except for children. All CCVA methods identified HIV/AIDS as the leading COD and provided a reasonable estimate (0.17–0.21) of the CSMF for HIV/AIDS compared with PCVA (0.22). InterVA-5 and InSilicoVA assigned a higher proportion of deaths due to TB compared to PCVA, suggesting that results for the combined categories of HIV/AIDS and TB should be reported when using CCVA.

All algorithms generated similar rankings of the COD (>0.60) using the rank correlation coefficient, except for neonates and children. The apparently contradictory measures of agreement in the neonates (high CSMF accuracy and low rank correlation) can be accounted for by PCVA assigning all neonatal deaths to only one of the two applicable causes for neonates (perinatal conditions and stillbirth) for the 25-cause list, while the CCVA algorithms assigned the majority to both applicable conditions with a few assigned to scattered causes that generally apply to older ages. A very high proportion of the causes are ‘perinatal’ leaving only a small number of cases to be considered for chance correlation in the kappa calculation. Although the kappa is negative, it is effectively zero, indicating that there was no correspondence between the PCVA and CCVA for the few cases that were not considered ‘perinatal’ on both.

Differences in COD assignment to diabetes, COPD, other cardiac disease and digestive and lung cancer between the different algorithms need further consideration to inform the revision of the SCI. The performance of all the algorithms as assessed using sensitivity was less than 60% for most causes, except HIV/AIDS, transport accidents, homicide (except Tariff 2.0) and suicide (except InterVA-5). The poorer performance of InterVA-5 in identifying suicide and Tariff 2.0 in identifying homicide requires further investigation to inform the revision of the SCI.

These findings align with other studies on CCVA, indicating that algorithms can identify COD distribution at the population level when compared with PCVA, but performance at the individual level is less satisfactory. Byass et al. [32] demonstrated that InterVA-5 detected HIV/AIDS related deaths with a similar specificity (90%) to that found in this study 93.3% (data not shown). McKormick et al. [9] found that InSilicoVA performs similarly to InterVA-5 at the population level, as found in this study. Jha et al. [33] reported that InterVA-5 had an 80% CSMF accuracy rate with PCVA for adults versus 84.0% found in this study. InSilicoVA-NT (NT- no training)^1^^1^The term InSilicoVA-NT was used by Jha et al [33] to differentiate the standard InSilicoVA which uses InterVA conditional probabilities and does not require a training dataset, from a version of InSilicoVA where a training dataset replaced the inbuilt conditional probabilities. [33] (a term used by Jha et al to denote the standard InSilicoVA using InterVA-5 conditional probabilities as used in our study), had a 77% concordance (CSMF accuracy) rate versus 84.0% found in this study. However, the performance at the individual level was inadequate and there could be implications for priority setting and resource allocation for specific causes, such as diabetes mellitus and cancers, without additional cause information from alternative data sources such as hospital admissions and deaths.

For implementation of VA into the routine vital statistics system in South Africa, especially at individual level, improving the SCI used by the algorithms is essential. Various initiatives, such as the creation of a vast reference archive of VA records with PCVA-assigned causes and the integration of narrative information and data from Minimally Invasive Tissue Sampling, have been proposed to achieve this.

## Limitations

Whilst the low recruitment rate of next-of-kin may introduce bias into the overall COD profile, it is unlikely to impact agreement rates when adjusted for chance. Unfortunately, it is not possible to predict what the likely bias could be from the available information. Additionally, whilst there is debate about validation methods and what constitutes the gold standard [33], the validity of the reference standard used in this study (PCVA COD) was not verified against medical records for this objective of the NCODV 2017/18 study. However, we feel that this is mitigated to a certain extent by the fact that clinicians had clinical experience in the public health system, the training and competency testing they received and the quality assessment process. In addition, after the consensus process there was almost 90% inter-reviewer agreement on the assigned COD. The MCCD training emphasized avoiding the use of immediate or intermediate causes of death, such as renal failure, without an underlying cause of death. This could have influenced the agreement with renal failure which is a cause on the VA cause list.

The assessment was based on a 25-cause list due to the limited sample size. We used meaningful groupings of related causes to reduce the number of causes with small numbers. While the 25-cause list spans an important range of common conditions, we were unable to assess the performance of the algorithms for conditions with small numbers. Although the sensitivity of CCVA for maternal deaths was around 50% and the positive predictive power for some of the algorithms was around 80%, there were insufficient cases to assess the performance of the algorithms for specific maternal COD (Ectopic pregnancy, abortion-related death, pregnancy-induced hypertension, obstetric haemorrhage, obstructed labour, pregnancy -related sepsis, anaemia of pregnancy, ruptured uterus and other and unspecified maternal causes).

## Conclusions

Although CCVA may reduce the costs associated with processing VA interviews by eliminating the need for costly PCVA, this study highlights significant potential for improving the algorithms for use in South Africa. An evaluation of whether the symptom-cause information matrix of CCVA can be improved is required. The feedback from physician reviewers in this study highlights the importance of the free text narrative for determining the underlying COD. Evaluating the incorporation of narrative information into the decision algorithms to potentially improve CCVA performance both at individual and population level should be a research priority. However, given the level of HIV/AIDS underreporting as a COD in South Africa, even the current CCVA methods provide a more accurate estimate of HIV/AIDS deaths than the official vital statistics and as such are extremely helpful for informing the national disease burden.

## Supplementary Material

Supplemental Material

## Paper context

Increasingly national-scale verbal autopsy implementations rely on cause-coding algorithms to automate coding of verbal autopsies to inform national burden of disease metrics. This comparison of the performance of InterVA-5, inSilicoVA and Tariff 2.0 against a reference PCVA dataset, which included community deaths, has corroborated the urgent need to improve the performance of these algorithms. A standard method for comparison of cause-coding algorithm performance and standard, freely available datasets for testing and validation are warranted.
