# Supplementary material for: Agreement between cause of death assignment by computer-coded verbal autopsy methods and physician coding of verbal autopsy interviews in South Africa
Source: Glob Health Action. 2023 Dec 1;16(1):2285105. doi: 10.1080/16549716.2023.2285105 (PMC10795603; doi:10.1080/16549716.2023.2285105)
Supplement: Supplemental Material [file ZGHA_A_2285105_SM6839.pdf]

NBD shortlist code	NBD shortlist title	VA code	VA title	ICD-10 codes
1	Pneumonia	102	Acute respiratory infection, including pneumonia	J00 - J22
2	HIV/AIDS	103	HIV/AIDS related death	B20 - B24
3	Diarrhoea	104	Diarrhoeal diseases	A00 - A09
4	Tuberculosis	109	Pulmonary tuberculosis	A15 - A16; U51 - U52
5	Other infectious diseases	105	Malaria	B50 - B54
		101	Sepsis	A40 - A41
		106	Measles	B05
		107	Meningitis and encephalitis	A39; G00 - G05
		108	Tetanus	A33 - A35
		110	Pertussis	A37
		111	Haemorrhagic fever	A92 - A99
		112	Dengue fever	A91
		199	Other and unspecified infectious disease	A17 - A19; A20 - A38; A42 - A44; A46; A48 - A89; B00 - B19; B25- B49; B55 - B99
6	Maternal	901	Ectopic pregnancy	O00
		902	Abortion-related death	O03 - O08
		903	Pregnancy-induced hypertension	O10 - O16
		904	Obstetric haemorrhage	O46; O67; O72
		905	Obstructed labour	O63; O66
		906	Pregnancy-related sepsis	O85
		907	Anaemia of pregnancy	O99
		908	Ruptured uterus	O71
		999	Other and unspecified maternal cause	O01 - O02; O20 - O45; O47 - O62; O68 - O70; O73 - O75; O76 - O84; O86 - O98
7	Perinatal	1001	Prematurity	P05 - P07
		1002	Birth asphyxia	P20 - P22
		1003	Neonatal pneumonia	P23 - P25
		1004	Neonatal sepsis	P36
		1005	Neonatal tetanus	A33
		1006	Congenital malformation	Q00 - Q99
		1099	Other and unspecified perinatal cause of death	P00 - P04; P08 - P15; P26 - P35; P37 - P94; P96
8	Digestive cancer	202	Digestive neoplasms	C15 - C26
9	Respiratory cancer	203	Respiratory neoplasms	C30 - C39
10	Other cancers	204	Breast neoplasms	C50
		205	Female reproductive neoplasms	C51 - C58
		206	Male reproductive neoplasms	C60 - C63
		299	Other and unspecified neoplasms	C07 - C14; C40 - C49; C60 - D48
11	Malnutrition	302	Severe malnutrition	E40 - E46
12	Diabetes mellitus	303	Diabetes mellitus	E10 - E14
13	Acute cardiac disease	401	Acute cardiac disease	I20 - I25
14	Stroke	402	Stroke	I60 - I69
15	Other cardiac disease	403	Sickle cell with crisis	D57
		499	Other and unspecified cardiac disease	I00 - I09; I10 - I15; I26 - I52; I70 - I99
16	Chronic obstructive pulmonary	501	Chronic obstructive pulmonary disease (COPD)	J40 - J44
17	Renal failure	701	Renal failure	N17 - N19
18	Other NCDs	301	Severe anaemia	D50 - D64
		502	Asthma	J45 - J46
		601	Acute abdomen	K35 - K37; K40 - K46; K56; R10
		602	Liver cirrhosis	K70 - K76
		801	Epilepsy	G40 - G41
		9800	Other and unspecified non-communicable disease	D55 - D89; E00 - E07; E15 - E35; E50 - E90; F00 - F99; G06 - G09; G10 - G37; G43 - G47; G50 - G99; H00- H95; J30 - J39; J47 - J99; K00 - K31; K35- K38; K40 - K93; L00 - L99; M00 - M99; N00- N16; N20 - N99; R00 - R09; R11 - R94
19	Stillbirth	1100	Stillbirths	P95
20	Transport accidents	1201	Road traffic accident	V01 - V89
		1202	Other transport accident	V90 - V99
21	Other accidents	1203	Accidental fall	W00 - W19
		1204	Accidental drowning and submersion	W65 - W74
		1205	Accidental exposure to smoke, fire and flames	X00 - X19
		1206	Contact with venomous animals and plants	X20 - X29
		1207	Accidental poisoning and exposure to noxious substa	X40 - X49
22	Homicide	1208	Intentional self-harm	X60 - X84
23	Suicide	1209	Assault	X85 - Y09
24	Other and unspecified external cause	1210	Exposure to force of nature	X30 - X39
		1299	Other and unspecified external cause of death	S00 - T99; W20 - W64; W75 - W99; X50 - X59; Y10 - Y98
25	Undetermined	9900	Cause of death unknown	R95 - R99

‘ VA – verbal autopsy
